# Parent Perspectives on Participation in Family-Centered Rounds and Informational Resource Use

**DOI:** 10.3389/fped.2020.00343

**Published:** 2020-06-30

**Authors:** Alexander F. Glick, Michael Goonan, Jacob Sherman, Diana Sandmeyer, Gabrielle Gold-von Simson

**Affiliations:** Department of Pediatrics, New York University School of Medicine/NYU Langone Health, New York, NY, United States

**Keywords:** patient engagement, hospital medicine, inpatient unit, pediatrics, communication, family-centered care

## Abstract

**Objectives:** Family-centered rounds (FCR) can improve communication and patient/family engagement. While use of informational resources (e.g., tablets, computers on wheels, paper notes) can guide FCR, there are limited data concerning parental perspectives on how use of these resources during FCR, or other factors, affect their engagement. Our objectives were to examine parental perspectives on factors that affect their participation during FCR and preferences for informational resources used.

**Methods:** We performed a cross-sectional study with English-speaking parents (*n* = 200), of pediatric inpatients at an academic medical center, present during FCR. We surveyed parents to ascertain factors they believed affect their engagement during FCR. We asked about their preferences regarding informational resources used by the medical team. Responses were analyzed using descriptive statistics. Parents described their reasoning behind resource preferences, and we categorized these responses.

**Results:** Parents reported that participation was affected by: clarity of the medical team's explanations (78.5%), understanding the information (75.5%), the child's health (74.5%), and being asked for their input (71%). Few (25%) parents believed the informational resource affects participation. Tablets were the preferred resource (24%) due to portability and ease of use, although 56% of parents had no preference.

**Conclusions:** Parents of hospitalized children placed importance on delivery of clear information and an “invitation” to participate during FCR. The resource used by the team was less important, although tablets were most preferred. Next steps are to examine factors associated with objective measures of participation and further study FCR in families with limited English proficiency.

## Introduction

Family-centered rounds (FCR) are daily, multidisciplinary rounds that include patients and their families as active participants in the discussion and medical decision-making process (1). FCR is currently the most common rounding model in pediatric inpatient units, particularly at academic institutions; it has been recognized as a best practice by the American Academy of Pediatrics as a means to increase family involvement (2). While all families may not choose to participate in FCR, this model allows for role modeling for trainees, enhances family involvement, and enriches a family's understanding of the plan (2, 3). Furthermore, greater patient and family engagement in the creation of individualized care plans improves health outcomes and satisfaction, making family participation during FCR a priority in delivery of quality care (4). While prior studies have established best practices for FCR, examined parent and provider perspectives on important components of rounding encounters, and examined factors associated with parent presence during FCR (3, 5–8), few studies have begun to quantify which of these factors parents believe may impact their engagement in the rounding process.

The informational resources (such as tablets, computers on wheels, and paper notes) used by medical teams to guide presentations during FCR may impact parental engagement, although parental resource preferences or views on which resources may impact their engagement have not been studied. Some studies suggest that computers, and specifically the electronic health record, may lead to better parent engagement by allowing providers to share information and assist physicians in making medical decisions (8, 9). However, use of computers during encounters may limit eye contact and create a barrier between the provider and family (10). Tablets may provide a solution to the physical barrier caused by larger computers on rounds (10, 11). Prior work has not specifically examined parent perspectives on use of these technology-based informational resources, or low-tech options such as paper-based notes, on their engagement in FCR.

The purpose of this study was to begin to examine which factors parents believe may affect their participation during FCR on an acute care inpatient pediatric unit. In addition, we examined parent preferences for the informational resource (tablet, computer on wheels, or paper notes) used by medical teams during FCR and explored reasons for their preferences. We hypothesized that parents would value a variety of factors that may impact their engagement during FCR. In addition, we hypothesized that parents would have a preference for use of tablets as an informational resource.

## Methods

### Study Design

This was a single-center, cross-sectional survey study. The survey consisted of questions related to two areas: (1) parental views on factors that impact their engagement in FCR and (2) resources used by the medical team during FCR. STROBE guidelines (12) were used in reporting this study. This study was approved by the New York University School of Medicine Institutional Review Board (s17-00586).

### Participants and Setting

This study examined parents of children who were hospitalized on the pediatric hospital medicine service on the acute care medical/surgical unit at an urban, tertiary care children's hospital within a university affiliated medical center from October 24, 2017 to November 30, 2018. We included English-speaking parents or legal guardians who were available and present for FCR. We excluded parents who were under 18 years of age or those who were previously enrolled. We focused on English-speaking parents, who represented >90% of families on the pediatric hospital medicine service and for whom FCR occurred in a more standardized fashion among providers at the study site, as a first step to optimize engagement for the majority of families.

The pediatric hospital medicine service conducts daily FCR on all patients (average: ~8 per day, range 4–15) each morning. The care team typically consists of a pediatric hospitalist (an attending general pediatrician who specializes in the care of hospitalized children), one third year resident, two interns, a sub-intern, three clerkship students, the bedside nurse, a clinical pharmacist, and the parent (should they choose to participate); subspecialty consultants are generally not a part of the rounding team. Parents are oriented to FCR as part of a welcome booklet and by members of the care team, who ask parents daily if they would like to attend FCR. Interns, sub-interns, and clerkship students present the patients, usually utilizing tablets, paper notes, or a computer on wheels as a resource; the senior resident also typically brings a computer on wheels to FCR to enter orders. After FCR, the medical team typically provided parents a written summary of the plan of care for the day.

### Survey

#### Survey Development

In order to inform the development of our survey, we first performed a literature review of prior work that examined potential factors that may impact parental engagement in FCR from both patient/family and provider perspectives (8, 10, 13). Once the initial list of potential factors that may affect engagement was determined, we held an informal focus group with 15 members of our institution's Family Advisory Council (FAC): parents whose children have been admitted to the hospital and have experienced FCR at our institution. This session was held during one of their regular meetings. Members of the FAC were not enrolled in the study but instead helped to determine which factors identified via literature review, and others they helped to identify through an open-ended discussion, would be included in our survey. The FAC did not recommend eliminating any variables but suggested adding two items to the list of factors (the length of hospitalization and the health of the child; see Appendix for full survey and list of factors). We then piloted our survey with 15 members of the FAC in which they informed our study team how they interpreted the meaning of these questions (i.e., cognitive interviews); FAC members deemed the final version of the survey questions to be understandable and complete in terms of content.

#### Enrollment and Survey Administration

On weekdays, trained research assistants (RAs) consecutively approached parents after the medical team discussed their child during FCR. Parents were approached once on the first weekday that they were present for FCR. RAs used a screening questionnaire to determine whether the parent met eligibility criteria (see section Participants and Setting).

RAs obtained verbal informed consent from parents who met eligibility criteria and agreed to participate; only verbal consent was required as surveys were anonymous, no protected health information was collected, and no medical records were reviewed. One parent was enrolled per child; when more than one parent was present, participation was self-determined.

RAs administered the survey to study subjects one time, immediately after enrollment. RAs read the survey questions and answer choices aloud to study subjects, who also followed along with a copy of the survey in front of them (see Appendix for full survey).

#### Outcomes

Our primary outcome was parent perspectives on factors affecting their participation and engagement during FCR. To assess this, we asked, “Which of the following affects how much you participate during rounds?” Participation was defined for parents as, “Ask questions, provide additional information, correct the medical team, or help in decision making,” to distinguish participation and engagement from simply attending FCR. RAs read each of the 12 possible options aloud one at a time; parents responded to each option individually and could report additional answers not listed as an open-ended response in order to ensure all potential ideas were expressed even if not present as answer choices on the survey.

A secondary outcome of interest was parent preference for resource (choices included tablet, paper notes, computer on wheels, other, no preference) used by the individual (typically a resident or medical student) presenting the patient during FCR. We also asked about the parent's least preferred resource. To assess the reasoning for their preferences, parents were asked an open-ended question (“why?”) after reporting their preferred and least preferred resource; RAs asked clarifying questions when needed and recorded parents' responses verbatim.

For those parents who could recall the resource used by the individual presenting during FCR, we asked if they believed the resource affected their understanding or participation during FCR (yes/no).

#### Additional Variables

We asked parents about their gender, age, education level, race, ethnicity, and prior experience attending FCR. We also asked for the child's age, gender, and length of stay. RAs recorded the type of informational resources used by the individual presenting during FCR.

### Analysis

Survey data for quantitative questions (yes/no, multiple choice) were analyzed using descriptive statistics. We also tallied the responses for additional factors that might impact parent engagement in the rounding process.

For open-ended question responses for parents' reasoning behind their most and least preferred resource, two members of the study team assessed their responses and categorized them thematically. Disagreements were adjudicated by consensus discussion.

Finally, for parents who had a preferred resource, we assessed the association between whether a parent experienced a resource during FCR (predictor) and whether a parent had a preference for use of that resource (outcome) using Fisher Exact Tests. We also performed an exploratory analysis using Fischer Exact and Chi Square Tests to determine if other parent or child characteristics (see section Additional Variables) were associated with preference for use of specific resources.

Our sample size of 200 parents was based on preliminary analyses of another component of this study: actual parent participation during FCR. Preliminary rounding observations showed that tablets were used during 60% of rounding encounters (compared to 40% of other resources). A sample size of 200 parents would allow for detection of a 20% difference in participation in groups exposed to a tablet compared to other resources (50 vs. 30% participation, alpha 0.05, power 0.8).

## Results

### Sample Characteristics

We approached 247 unique parents during the study period. A total of 200 were enrolled and completed the full survey (see Supplemental Figure 1 for more details on participant eligibility and refusals).

Table 1 reports information on parent and child demographics. The individual presenting the patient used the following informational resources (several used more than one resource): tablets (60.5% of encounters), paper notes (64%), computer on wheels (2.5%), and no resource (2.5%).

**Table 1:** Parent and child characteristics (*n* = 200).

**Characteristic**	**Value**
Parent	
Age in years, median (IQR)	36 (31.25–42)
Female, *n* (%)	158 (79)
Race/ethnicity	
White, non-hispanic, *n* (%)	105 (52.5)
Hispanic, *n* (%)	43 (21.5)
Asian, non-hispanic, *n* (%)	21 (10.5)
Black, non-hispanic, *n* (%)	20 (10)
Other, *n* (%)	10 (5)
Refused, *n* (%)	1 (0.5)
Education	
Did not complete high school, *n* (%)	14 (7)
High school diploma or equivalent	46 (23)
Some college (without Bachelor's degree)	47 (23.5)
Bachelor's degree	46 (23)
Education beyond Bachelor's degree	47 (23.5)
Prior experience attending family-centered rounds	88 (44)
Child	
Age in months, median (IQR)	24 (5–108)
Male, *n* (%)	103 (51.5)
Length of Stay in days, median (IQR)	2 (1–4)

*IQR, interquartile range*.

### Factors Affecting Participation in Family-Centered Rounds

The majority of parents reported that their participation during FCR was influenced most when explanations from the medical team were clear (78.5%), when they understood the medical information (75.5%), based on the health of the child (74.5%), and when the medical team asked for their input (71%) (Table 2). Fewer parents believed that the type of resource used affected their participation (25%). Additional factors that parents suggested impacted their participation included if they perceived information they had received to be “conflicting,” if they felt they were not being heard, if they had specific questions for the team, if subspecialists and consulting physicians were present, the timing of FCR, and the child's age.

**Table 2:** Parent perspectives on factors impacting participation in family-centered rounds (*n* = 200).

**Factor (phrasing used in parent survey)**	**Parent indicated positive response, *n* (%)**
Whether the medical team explains things in a way that is easy to understand	157 (78.5)
How well you understand the medical information	151 (75.5)
The health of your child	149 (74.5)
If the medical team asks for your input	142 (71.0)
Eye contact from the medical team	127 (63.5)
The amount of time the medical team spends with you	127 (63.5)
The relationship you have with your child's doctor	123 (61.5)
Level of experience of the medical team	109 (54.5)
How long you have been in the hospital	98 (49.0)
The type of resources (such as a tablet, computer on wheels, or paper notes) used by the medical team	50 (25.0)
The size of the medical team on rounds	50 (25.0)

### Preferred and Least Preferred Informational Resources, Reasons, and Associated Variables

**Figure 1:**
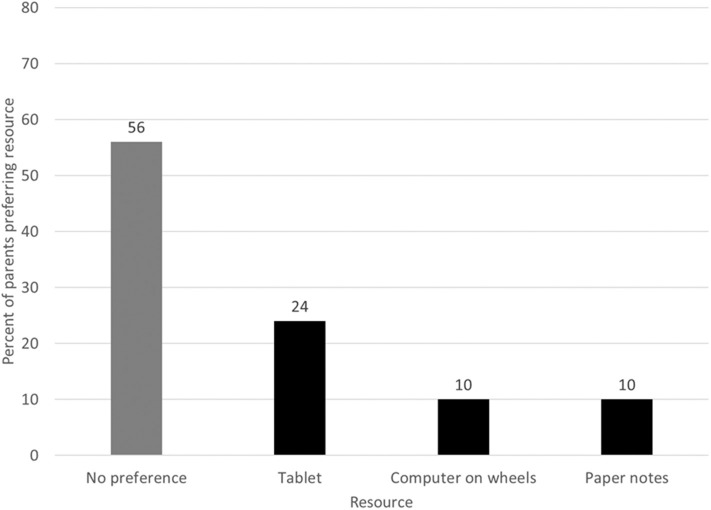
Parent resource preferences during family-centered rounds (*n* = 200).

The majority of parents (56%) had no preference for the informational resource used by the medical team (Figure 1). Table 3 categorizes the common themes that emerged for reasoning behind the parents' preferences from their perspective. Most parents believed that the resource used does not affect information delivery and should be the provider's choice. When parents *did* have a preference, tablets were the most commonly preferred resource (24% of all parents). Parents commented that tablets allowed for easy information access and sharing, were smaller in size, and had up-to-date information.

**Table 3:** Major themes identified related to parent reasoning for preferences for informational resources to be used during family-centered rounds.

	**Tablet**	**Computer on wheels**	**Paper notes**	**No preference**	Cell phones^*^
Reasons for preferring resource	• Ease of use and access, convenience, organization • Portability • Ease of information sharing	• Ease of information access and sharing • Personal preference • Ease of documentation and speed	• Personal preference or distrust of technology • Easier to access and document information • Easier to understand	• Does not affect care or information delivery • No reason • Should be provider preference	N/A
Reasons for not preferring resource	• Easy to lose • Information not accurate slow	• Large, diminishes eye contact • Information not accurate • Difficult to access information	• Not easy to share or document information • Information not accurate or secure, easy to lose • Messy, old-fashioned, slow	• No reason • Does not affect care or information delivery • Should be provider preference	Not professional or secure

* *Mentioned as an “other” option by two parents for least preferred resource*.

Of parents with a resource preference, those who were exposed to tablets during FCR were more likely to have a resource preference for tablets compared to those who were not (63.5 vs. 36.4%, *p* = 0.03). Exposure to computers on wheels or paper notes during FCR were not associated with preference for these resources. No other parent or child characteristics were associated with preference for a specific resource.

A greater majority of parents (76.5%) did not identify a least preferred resource. Paper notes (13%), computers on wheels (7.5%), tablets (2%), and cell phone (1%, given as a free response answer by two parents) were identified as the least preferred resource by fewer parents. Table 3 summarizes the reasons parents reported for these choices.

### Impact of Informational Resource on Understanding and Participation

A total of 86.5% of parents could recall the primary resource utilized by the medical team during FCR. Of this subgroup, 77.5% believed that the resource did not affect their understanding of the information presented regardless of what resource was used, and 88.4% believed that the resource used did not affect their participation.

## Discussion

In our study, the factors that parents thought most significantly impacted their engagement during FCR were the medical team delivering information in a clear manner, an invitation to actively participate in the rounding process, and the health of the child. Most parents did not believe that the type of informational resource impacted their participation or understanding. Of those parents who did have a preference, tablets were most frequently favored.

While prior studies have emphasized the importance of clear communication with families during FCR (5, 6, 14, 15), our study demonstrated that parents believe these factors also impact their engagement in the rounding process. One prior study showed, however, that fewer than half of parents understood the plan of care discussed during FCR (16). Use of health literacy-informed communication strategies such as teachback, or explicitly checking understanding by having families restate information in their own words, leads to improved learning in other settings (17, 18). Pediatricians should utilize such communication strategies during FCR, as well as during other types of encounters with patients and families.

Inviting the family to participate was another factor most parents believed affects their engagement. One prior study demonstrated that an FCR bundle that included an explicit invitation for the patient/family to speak was associated with a decrease in preventable adverse events and improved family engagement (19). Prior studies, however, have not specifically isolated the impact of inviting parents to participate on their actual engagement. Future work should examine whether an explicit invitation to share information, among other factors, impacts objective measures of engagement. Even when families are invited to participate in discussions during FCR, they may still feel reluctant to do so (20). Pediatricians can help overcome this barrier by creating an environment where parents feel like they can ask questions and interrupt without feeling judged (20–22); pediatricians should ensure that they actively invite and encourage participation for interested parents.

We also demonstrated that parents believed that the health of their child affects their engagement during FCR—although we did not explore whether parents were more likely to engage when their children were more or less sick. Prior studies in the pediatric intensive care unit have found inconsistent associations between disease severity and attendance on rounds (6, 7). Additional research is needed to explore the impact of illness severity in acute care settings, outside of the intensive care unit, in regards to parents' presence and engagement during FCR in order to build on the preliminary findings of our survey.

Our study found that a majority of parents believed that the type of informational resource used by the medical team does not impact their participation or understanding of information discussed during FCR. Tablets were favored by parents who had a preferred resource, especially those who saw tablets used during FCR. Other inpatient studies have shown that tablet use can increase engagement, satisfaction, and understanding (23, 24), though this has not previously been studied in the context of FCR. It is possible that providers may be simply using tablets as resources for themselves, as opposed to as a tool to increase engagement. Pediatricians can try to use these resources to maximize engagement, for example by sharing laboratory results. Parents may have been less likely to choose a preferred resource if not exposed to it. A deeper analysis of parental preferences in regard to which informational resources would be used in which circumstances is warranted. Our analyses also did not find associations between parent or child characteristics and resource preference, although our study was likely not powered to detect a statistical difference. It is possible that certain groups may have specific preferences, which can be the focus of future work.

While our study identified factors that most parents agreed would impact their engagement, one FCR model may not work for all families. Patient and family-centered care should take into account the individual preferences of patients and families (3, 8, 10). For example, some families may be intimidated by a larger medical team (10), even though not identified as a factor that may impact engagement by most families in our survey. Medical teams should make an effort to invite parents to attend and engage in FCR—yet must take individual parent and patient preferences into account when conducting FCR. Such preferences can be explored when orienting patients and families to FCR on admission.

Our study has limitations. Generalizability is limited as the study was conducted at a single site, on a single service, and only with English-speaking parents who were generally well-educated. In particular, families of children with limited English proficiency feel less empowered to participate during FCR (25), and provider communication with these families is often suboptimal compared to English-speaking families (16, 25). As the average length of stay was 2 days, it is possible that the findings may not be generalizable to those with longer length of stay, including children with chronic medical conditions; parents of medically complex children often have additional insights into their child's medical issues (26) and therefore may have more to add or differing perspectives on what would impact their engagement in FCR. Furthermore, we specifically focused on parents who attended FCR. It is likely that these families are more engaged at baseline compared to families who do not attend FCR; future work should focus on learning more about why some parents choose not to participate during FCR, even when invited, in order to determine better ways to engage them. This study assessed factors that impact participation during FCR from the parent's perspective; it is possible that parents' views may differ from the factors that are associated with more objective measures of participation. We also did not assess views of faculty, trainees, or other members of the care team on how to best engage families. The survey also did not examine parental views on the direction of impact on engagement (i.e., more or less engagement), and parents did not rank which factors they believed to be most important. Parents may have been biased to choose factors impacting their participation that were provided as answer choices; additional qualitative work is likely needed to further elucidate parental views on this issue. Our survey design, however, was informed by discussions with parents and prior research, and few parents suggested additional factors when asked. Finally, our study examined views of the parent and not the child, although children in our study were typically younger (median age 24 months old) and themselves not developmentally able to engage in the rounding process. It is possible, however, that older children and teenagers may themselves have viewpoints related to engagement in the rounding process and preferences for informational resources used that are different than their parents. For example, they may be more likely to be interested in use of technology as a tool for engagement during the rounding process.

Our findings underscore the value of clear communication and engaging discussion during FCR. Parents also valued the opportunity to be explicitly invited to participate in the discussion, which although recommended is not universally performed. Parents believed these factors impact their participation during FCR more so than the type of informational resource used by the medical team. Given that most parents either preferred tablets or deferred to the provider's preference for the informational resource to be used during FCR, as well as other potential benefits of tablets (e.g., convenience of use during FCR, portability, real-time electronic health record access), further study of the use of tablets fostering an engaging rounding experience may be worthwhile. Members of the rounding team can consider using tablets as an informational resource for themselves, as well as to share up-to-date information with the patient and family, without the physical barriers caused by larger computers. One must also consider that parents may have been more likely to prefer tablets given their more frequent exposure to this resource; additional studies in which parents are randomized to which type of resource they experience may be worthwhile.

Future work will include observations of FCR to determine factors associated with more objective measures of engagement and participation. We also hope to examine the perspectives of members of the rounding team on how to best engage families. Future work should also determine whether training pediatricians in how to invite parents to participate and maintain eye contact during FCR affects parental engagement. In addition, we will seek to optimize use of technology during the rounding process to maximize engagement of families while minimizing barriers to efficiency and potential sources of error. Finally, we will work to further study and improve the FCR process for families with limited English proficiency.

## Data Availability Statement

The datasets generated for this study are available on request to the corresponding author.

## Ethics Statement

The studies involving human participants were reviewed and approved by NYU School of Medicine Institutional Review Board. Written informed consent for participation was not required for this study in accordance with the national legislation and the institutional requirements.

## Author Contributions

AG conceptualized and designed the study, carried out the initial analyses, drafted the initial manuscript, and approved the final manuscript as submitted. MG and GG helped to conceptualize and design the study, assisted with data analysis, critically reviewed and revised the manuscript, and approved the final manuscript as submitted. JS helped to collect data, assisted with data analysis, helped to draft the initial manuscript, critically reviewed and revised the manuscript, and approved the final manuscript as submitted. DS helped to design and pilot test the survey, collected data, critically reviewed and revised the manuscript, and approved the final manuscript as submitted. All authors contributed to the article and approved the submitted version.

## Conflict of Interest

The authors declare that the research was conducted in the absence of any commercial or financial relationships that could be construed as a potential conflict of interest.

## Abbreviations

FAC: Family Advisory Council
FCR: Family Centered Rounds
RAs: Research Assistants.
