# Supplementary material for: Parent Perspectives on Participation in Family-Centered Rounds and Informational Resource Use
Source: Front Pediatr. 2020 Jun 30;8:343. doi: 10.3389/fped.2020.00343 (PMC7338563; doi:10.3389/fped.2020.00343)
Supplement: Supplementary file 1 [file Data_Sheet_1.docx]

**APPENDIX**

These first questions are about your experiences on rounds in general.

Before today, have you attended family centered rounds?

0 □ No

1 □ Yes

How often do you participate during rounds? By participate, we mean ask questions, provide additional information, correct the medical team, or help in decision making?

1 □ Never

2 □ Rarely

3 □ Sometimes

4 □ Often

5 □ Always

Which of the following affect how much you participate during rounds? We can check off all that apply.

1 □ How long you have been in the hospital

2 □ How well you understand the medical information

3 □ If the medical team asks for your input

4 □ Eye contact from the medical team

5 □ Level of experience of the medical team

6 □ The type of resources (such as a tablet, computer on wheels, or paper notes) used by the medical team

7 □ The amount of time the medical team spends with you

8 □ The relationship you have with your child’s doctor

9 □ Whether the medical team explains things in a way that is easy to understand

10 □ The size of the medical team on rounds

11 □ The health of your child

12 □ Other________________________

During rounds, there are several resources that the medical team uses to access and report information about your child. These include tablets (such as iPads), paper notes, and computers on wheels. The next set of questions will ask about your experience with these resources.

During rounds, which **one** of the following would you prefer that the medical team use as a resource when reporting information about your child?

1 □ A tablet, like an iPad

2 □ Paper notes

3 □ A computer on wheels

4 □ Other___________________________________________________

5 □ No preference

Why? _______________________________________

During rounds, which **one** of the following would you **not want** the medical team to use as a resource when reporting information about your child? [READ CHOICES ALOUD]

1 □ A tablet, like an iPad

2 □ Paper notes

3 □ A computer on wheels

4 □ Other___________________________________________________

5 □ No preference

Why? _____________________________________________________________

The next questions are about the resource(s) (such as a tablet, computer or paper notes) the doctor or student used today when talking about your child.

Which of these did the doctor or student use on rounds today? We can check off all that apply.

1 □ A tablet, like an iPad

2 □ Paper notes

3 □ A computer on wheels

4 □ Other___________________________________________________

5 □ No resource

6 □ Unsure

Which of these did the doctor or student use the most?

1 □ A tablet, like an iPad

2 □ Paper notes

3 □ A computer on wheels

4 □ Other___________________________________________________

Do you feel like the doctor’s or student’s use of [ABOVE RESOURCE] on rounds made what they told you...

1 □ Easier to understand

2 □ Harder to understand

3 □ Did not affect your understanding

Do you feel like the doctor’s or student’s use of [ABOVE RESOURCE] on rounds made you…

1 □ Participate more on rounds

2 □ Participate less on rounds

3 □ Did not affect your participation on rounds
