# Supplementary figures and images for: Parent Perspectives on Participation in Family-Centered Rounds and Informational Resource Use
Source: Front Pediatr. 2020 Jun 30;8:343. doi: 10.3389/fped.2020.00343 (PMC7338563; doi:10.3389/fped.2020.00343)

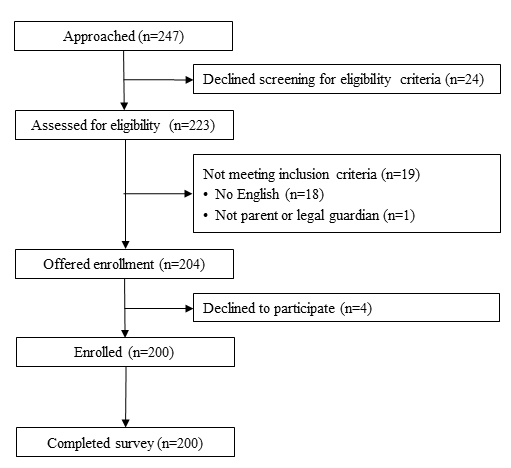

Supplement: Supplementary file 2 [file Image_1.TIF]
